# Stable complex formation of CENP-B with the CENP-A nucleosome

**DOI:** 10.1093/nar/gkv405

**Published:** 2015-04-27

**Authors:** Risa Fujita, Koichiro Otake, Yasuhiro Arimura, Naoki Horikoshi, Yuta Miya, Tatsuya Shiga, Akihisa Osakabe, Hiroaki Tachiwana, Jun-ichirou Ohzeki, Vladimir Larionov, Hiroshi Masumoto, Hitoshi Kurumizaka

**Affiliations:** 1 Laboratory of Structural Biology, Graduate School of Advanced Science and Engineering, Waseda University, 2-2 Wakamatsu-cho, Shinjuku-ku, Tokyo 162-8480, Japan; 2 Laboratory of Cell Engineering, Department of Frontier Research, Kazusa DNA Research Institute, 2-6-7 Kazusa-Kamatari, Kisarazu, Chiba 292-0818, Japan; 3 Development Therapeutic Branch, National Cancer Institute, National Institutes of Health, Building 37, Room 5040, 9000 Rockville Pike, Bethesda, MD 20892, USA

## Abstract

CENP-A and CENP-B are major components of centromeric chromatin. CENP-A is the histone H3 variant, which forms the centromere-specific nucleosome. CENP-B specifically binds to the CENP-B box DNA sequence on the centromere-specific repetitive DNA. In the present study, we found that the CENP-A nucleosome more stably retains human CENP-B than the H3.1 nucleosome *in vitro*. Specifically, CENP-B forms a stable complex with the CENP-A nucleosome, when the CENP-B box sequence is located at the proximal edge of the nucleosome. Surprisingly, the CENP-B binding was weaker when the CENP-B box sequence was located in the distal linker region of the nucleosome. This difference in CENP-B binding, depending on the CENP-B box location, was not observed with the H3.1 nucleosome. Consistently, we found that the DNA-binding domain of CENP-B specifically interacted with the CENP-A-H4 complex, but not with the H3.1-H4 complex, *in vitro*. These results suggested that CENP-B forms a more stable complex with the CENP-A nucleosome through specific interactions with CENP-A, if the CENP-B box is located proximal to the CENP-A nucleosome. Our *in vivo* assay also revealed that CENP-B binding in the vicinity of the CENP-A nucleosome substantially stabilizes the CENP-A nucleosome on alphoid DNA in human cells.

## INTRODUCTION

During mitotic cell division, microtubules are attached to kinetochores formed on each chromosome, to direct the segregation of sister chromatids into daughter cells (1–3). In chromosomes, the centromere is an important region that provides kinetochore assembly sites (4,5). CENP-A, an evolutionarily conserved histone H3 variant, is an essential protein component for functional centromeres (6–10). Genetic and cell biological studies revealed that CENP-A depletion induces chromosome missegregation, due to improper centromere formation (8,10–14).

CENP-A forms a complex with histones H2A, H2B and H4, and the DNA is wrapped around the CENP-A-H2A-H2B-H4 complex (5). The crystal structure revealed that CENP-A, H2A, H2B and H4 form the CENP-A nucleosome, with a histone octamer containing two each of CENP-A, H2A, H2B and H4 and the DNA left-handedly wrapped around it (15). Consistently, biochemical and biophysical experiments confirmed the formation of the CENP-A nucleosome as the histone octamer (16–20). The octameric nucleosomes containing CENP-A and its homologues were also found in yeast, fly, and human cells (21–25). On the other hand, the hemisome, consisting of one each of CENP-A, H2A, H2B and H4, has been proposed as another form (26–28), which may appear in a cell cycle-dependent manner (29,30).

Among the centromeric proteins, CENP-B (6,31–33), CENP-C (6,34,35), CENP-S (36–40), CENP-T (36–40), CENP-W (38–40) and CENP-X (37,39,40) are known to possess DNA-binding activity. CENP-B recognizes and binds to the 17 base-pair CENP-B box DNA sequence, and is the only sequence-specific DNA binding protein in mammalian centromeres (32,33,41). A micrococcal nuclease (MNase) mapping experiment revealed that the CENP-B box DNA is preferentially located near the entry/exit sites of the CENP-A nucleosome *in vitro* (42). This is quite consistent with the following observations. The CENP-B N-terminal DNA binding domain (CENP-B DBD) is required for the *de novo* assembly of CENP-A on input naked alphoid DNA (43). CENP-A nucleosomes are phased at the CENP-B boxes on alphoid DNA (23,25,44), and the CENP-A N-terminal tail is required for kinetochore function through CENP-B (45). However, we did not detect an obvious difference in CENP-B binding between the CENP-A and H3.1 nucleosomes in our previous *in vitro* analysis (46). These discrepancies were intriguing, and we realized the importance of investigating the structural and functional properties of the interactions between the CENP-A nucleosome and CENP-B on alphoid DNA, both *in vivo* and *in vitro*.

In the present study, we reconstituted the CENP-A and canonical H3 nucleosomes complexed with the DNA-binding domain of CENP-B (CENP-B DBD), and found that the CENP-B bound to the CENP-A nucleosome is stably retained in the presence of an excess amount of competitor DNA, in contrast to the CENP-B bound to the canonical H3 nucleosome. Surprisingly, the CENP-B bound to the proximal region of the CENP-A nucleosome was more stably retained, as compared to that bound to the distal linker region, suggesting that the CENP-A nucleosome accommodates CENP-B at the proximal region with high affinity. Consistently, we found that the CENP-B DBD specifically bound to CENP-A, but not to H3.1, *in vitro*. We also found that the CENP-B binding substantially stabilizes the CENP-A nucleosome on alphoid DNA in human cells.

## MATERIALS AND METHODS

### Purification of human CENP-B DBD, CENP-A and histones

Human histones (H2A, H2B, H3.1, CENP-A and H4) were expressed as N-terminally His_6_-tagged proteins in *Escherichia coli* cells (47), and purified by nickel-nitrilotriacetic acid (Ni-NTA) agarose chromatography (Qiagen), thrombin protease (GE Healthcare) treatment, and Mono S column chromatography (GE Healthcare), as described previously (15). The human CENP-B DBD was expressed in *E. coli* cells, and was purified as described previously (46). H3.1^CATD^ was produced as a bacterially expressed recombinant protein, using the modified H3.1 expression vector. In the H3.1^CATD^ expression vector, the DNA fragment encoding amino acid residues 75–112 of H3.1 was replaced by the corresponding CENP-A region, encoding amino acid residues 75–114. H3.1^CATD^ was then purified by the same method as for H3.1 (48).

### Preparation of the 166-bp human centromeric α-satellite repeat DNA

Eight tandem repeats of the 166 base-pair human α-satellite DNA containing the CENP-B box sequence were ligated into the pGEM-T easy vector (Promega), which was amplified in *E. coli* DH5α cells. The 166 base-pair DNA sequence with the proximal CENP-B box is as follows: 5′-ATCTATTTGGACCGCATTGAGGCCTTCGTTGGAAACGGGATTTCTTCATTTCATGCTAGACAGAAGAATTCTCAGTAACTTCTTTGTGCTGTGTGTATTCAACTCACAGAGTGGAACGTCCCTTTGCACAGAGCAGATTTGAAACACTCTTTTTGTAGTCGACGAT-3′. The 166 base-pair DNA sequence with the distal CENP-B box (-20) is as follows: 5′-ATCCTTCGTTGGAAACGGGAGGCTATCGTCTGCAGCGCCATTTCTTCATTTCATGCTAGACAGAAGAATTCTCAGTAACTTCTTTGTGCTGTGTGTATTCAACTCACAGAGTGGAACGTCCCTTTGCACAGAGCAGATTTGAAACACTCTTTTTGTAGTCGACGAT-’3. The CENP-B box sequences are underlined. The 166 base-pair DNA fragment was generated by EcoRV digestion, and was purified by TSK gel DEAE-5PW (TOSOH) anion exchange column chromatography.

### Preparation of the CENP-A, H3.1 and H3.1^CATD^ nucleosomes

The CENP-A, H3.1 and H3.1^CATD^ nucleosomes were reconstituted with the 166 base-pair satellite DNA fragment by the salt dialysis method, as described previously (15,46,48). The reconstituted nucleosomes were purified by preparative non-denaturing polyacrylamide gel electrophoresis (Prep Cell Model 491: Bio-Rad).

### Preparation of nucleosomes complexed with the CENP-B DBD

The purified CENP-A, H3.1, or H3.1^CATD^ nucleosomes (463 nM) were incubated with the CENP-B DBD (2.6–3.5 μM) for 20 min at 37ºC. The nucleosomes complexed with the CENP-B DBD were separated from the free nucleosomes and the CENP-B DBD by preparative non-denaturing polyacrylamide gel electrophoresis (Prep Cell Model 491: Bio-Rad). The purified nucleosome-CENP-B DBD complexes were concentrated and stored on ice.

### Competitive assay for CENP-B retention

The CENP-A, H3.1, or H3.1^CATD^ nucleosomes (122 nM) complexed with the CENP-B DBD were incubated with the 166-bp DNA containing the CENP-B box sequence in 8 μl of 28 mM Tris-HCl (pH 7.5) buffer, containing 1.1 mM dithiothreitol, 0.05 mM EDTA and 0.1 μg/μl bovine serum albumin (BSA). After an incubation at 25ºC for 20 min, the samples were fractionated by 6% non-denaturing polyacrylamide gel electrophoresis in 0.2 x TBE buffer at 8.3 V/cm for 1 hr, and were visualized by ethidium bromide staining.

### Pull-down assay

For the pull-down assay, the His_6_-tagged CENP-B DBD complexed with a 21 base-pair DNA was prepared as described previously (41). The DNA sequence used in the His_6_-tagged CENP-B DBD-DNA complex formation was 5′-GCC TTC GTT GGA AAC GGG ATT-3′, and the CENP-B box sequence is underlined. The purified CENP-A-H4, H3.1-H4, or H3.1^CATD^-H4 complex (50 nM) was mixed with the His_6_-tagged CENP-B DBD-DNA complex (50 nM), in a reaction mixture (1 ml) containing 20 mM Tris-HCl (pH 8.0), 300 mM NaCl, 60 mM imidazole, 0.3% Tween-20, 0.01 μg/μl bovine serum albumin, and nickel-nitrilotriacetic acid (Ni-NTA) agarose beads (6 μl, 50% slurry), at 4ºC for 60 min. The Ni-NTA agarose beads were then pelleted by centrifugation at 3000 r.p.m. for 7 min, and were washed three times with 0.5 ml of 20 mM Tris-HCl buffer (pH 8.0), containing 500 mM NaCl, 30 mM imidazole, and 0.3% Tween-20. The proteins captured by the Ni-NTA agarose beads were eluted with 1 M imidazole, and were analyzed by 16% SDS-PAGE with Coomassie Brilliant Blue staining.

### Cell lines and culture

The human fibrosarcoma HT1080 cell line stably expressing the tetR-EYFP-HJURP fusion protein was established by transfection of the tetR-EYFP-HJURP plasmid (49) with Fugene HD (Promega:E2311). Transfected cells were selected with 200 ng/μl Hygromycin B (Wako:084–07681). Cells were cultured in Dulbecco's Modified Eagle's medium (Wako:043–30085), supplemented with 10% tet-approved FBS (Clontech: 631106), at 37ºC in a 5% CO_2_ atmosphere.

### ChIP assay followed by real-time PCR quantification and competitive PCR

BAC DNAs containing synthetic alphoid^tetO^ DNAs with wild type CENP-B boxes and mutant CENP-B boxes (49; 50kb) (total 400 ng) and the Halo-CENP-A expressing plasmid (CENP-A inserted into pFN21A: Promega, G2821) (25 ng) were co-transfected into 70% confluent HT1080 tetR-EYFP-HJURP expressing cells in 6 well plates with Fugene HD. The transfected cells were cultured in medium containing 1 ng/μl doxycycline. At 24 hrs after transfection, to deposit CENP-A at the tetO sites on the introduced alphoid BAC DNAs, the cells were washed twice with PBS, and cultured in doxycycline-free medium for 24 hrs. To dissociate tetR-EYFP-HJURP from the tetO sites, doxycycline was added to the medium at a concentration of 1 ng/μl. The cells were trypsinized, harvested in a centrifuge tube, washed once with PBS, and fixed in 0.5% formaldehyde (Sigma: F8775) at 22°C for 10 min. The reaction was stopped by the addition of glycine to a final concentration of 125 mM. The cells were then incubated at room temperature for 5 min, and washed with PBS. The cells were suspended in sonication buffer, containing 20 mM Tris-HCl (pH 8.0), 0.5 mM EDTA, 1.5 μM aprotinin, 10 μM leupeptin, 1 mM DTT, 0.02% SDS and 40 μM MG132, and then sonicated with a Bioruptor (Cosmobio) to generate an average DNA size of 0.5∼1kb. After the sonication, the soluble chromatin (as input) was recovered by centrifugation, diluted with IP buffer, containing 20 mM Tris-HCl (pH 8.0), 300 mM NaCl, 0.5 mM EDTA, 1.5 μM aprotinin, 10 μM leupeptin, 40 μM MG132, 1 mM DTT, 0.05% SDS, 1% Triton X-100 and 5% glycerol, and immunoprecipitated using anti-CENP-A (A1: 5 μg), anti-CENP-B (5E6C1: 2 μg), anti-H3 (MA301A: 1 μg) and anti-GFP (also recognizing EYFP, Roche: 11814460001: 2 μg) monoclonal antibodies with Dynabeads-protein G (Life Technologies: 10003D). The DNA was purified from the immunoprecipitates and quantified by real-time polymerase chain reaction (PCR), using SYBR Premix EX taq II (Takarabio: RR820S) and the following primer sets: tetOF (5′-CTCTTTTTGTGGAATCTGCAAGTG-3′) and tetOR (5′-TCTATCACTGATAGGGAGAGCTCT-3′) for alphoid^tetO^ DNA.

After PCR, the competitively amplified wild type and mutant CENP-B box alphoid^tetO^ DNAs were digested by the endonuclease EcoRV. The digested samples were fractionated by agarose gel electrophoresis. Gel images were quantified using the Image J software (NIH).

## RESULTS

### CENP-B binds more stably to the CENP-A nucleosome than the H3 nucleosome

**Figure 1.**
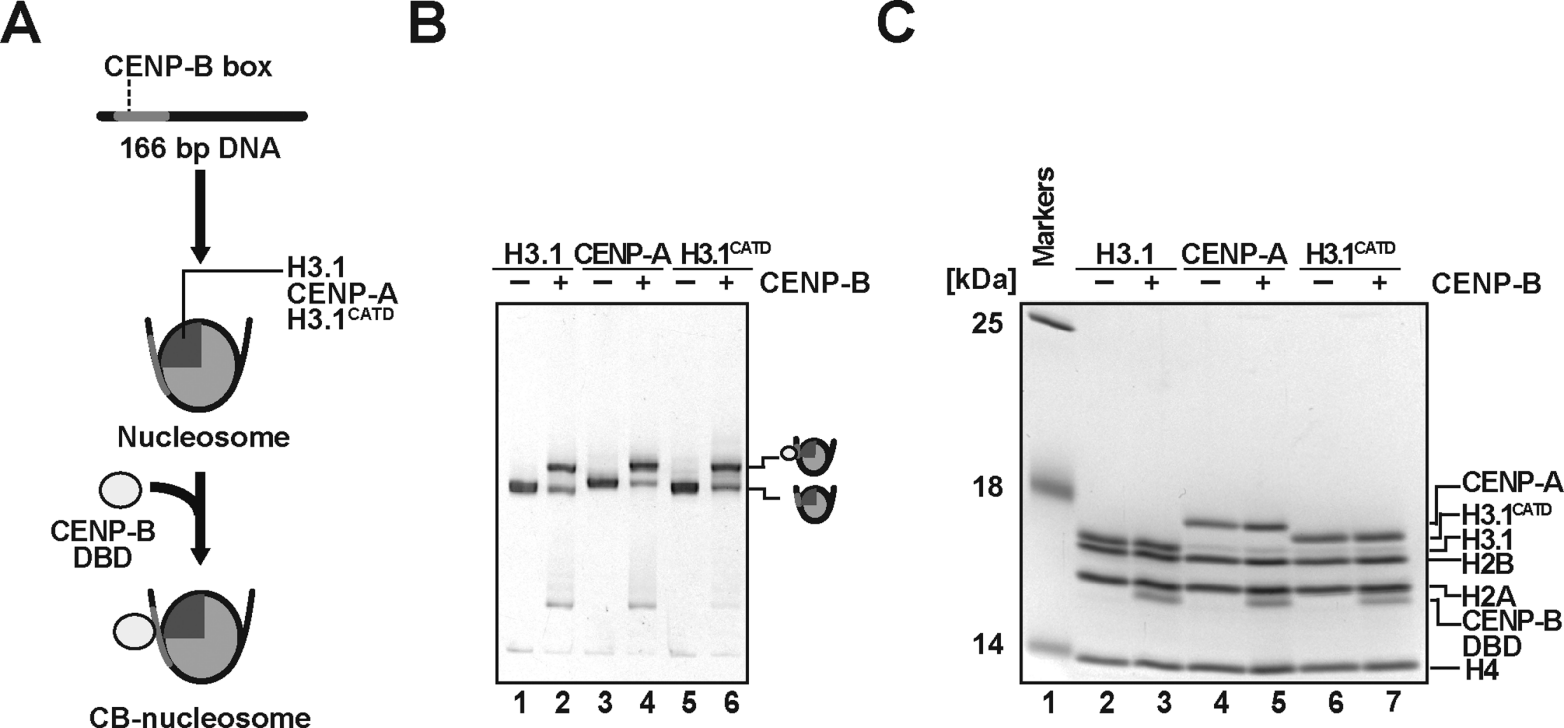
CENP-B binds to the CENP-A and H3.1 nucleosomes. (**A**) Schematic representation of CENP-B DBD binding to nucleosomes. (**B**) Electrophoretic mobility shift assay. The H3.1, CENP-A and H3.1^CATD^ nucleosomes (lanes 1, 3 and 5, respectively) and those complexed with the CENP-B DBD (lanes 2, 4 and 6, respectively) were analyzed by non-denaturing 6% polyacrylamide gel electrophoresis with ethidium bromide staining. (**C**) Protein contents of the H3.1, CENP-A and H3.1^CATD^ nucleosomes (lanes 2, 4 and 6, respectively) and those complexed with the CENP-B DBD (lanes 3, 5 and 7, respectively), analyzed by SDS-15% polyacrylamide gel electrophoresis with Coomassie Brilliant Blue staining.

CENP-B is known to bind to the nucleosomal CENP-B box sequence, if it is located near the entry/exit sites of nucleosomes (42,46). Therefore, we reconstituted nucleosomes containing either CENP-A or H3.1, and prepared the CENP-A and H3.1 nucleosomes complexed with the CENP-B DBD, composed of the N-terminal 129 amino acid residues of CENP-B (44,50). A human α-satellite DNA fragment with a CENP-B box sequence was used for the nucleosome reconstitution (Figure 1A). Previous MNase analyses confirmed that the CENP-B box sequence was located near an entry/exit (proximal) site of nucleosomes with this DNA sequence (42,46). The reconstituted CENP-A and H3.1 nucleosomes with the CENP-B DBD (CA-CB nucleosome and H3.1-CB nucleosome, respectively) were purified by native polyacrylamide gel electrophoresis, using a Prep Cell apparatus (Bio-Rad) (Figure 1B). The purified CA-CB or H3.1-CB nucleosomes contained the CENP-B DBD with histones H2A, H2B, H4 and CENP-A or H3.1, respectively (Figure 1C, lanes 3 and 5). These results indicated that the CA-CB and H3.1-CB nucleosomes were properly prepared.

**Figure 2.**
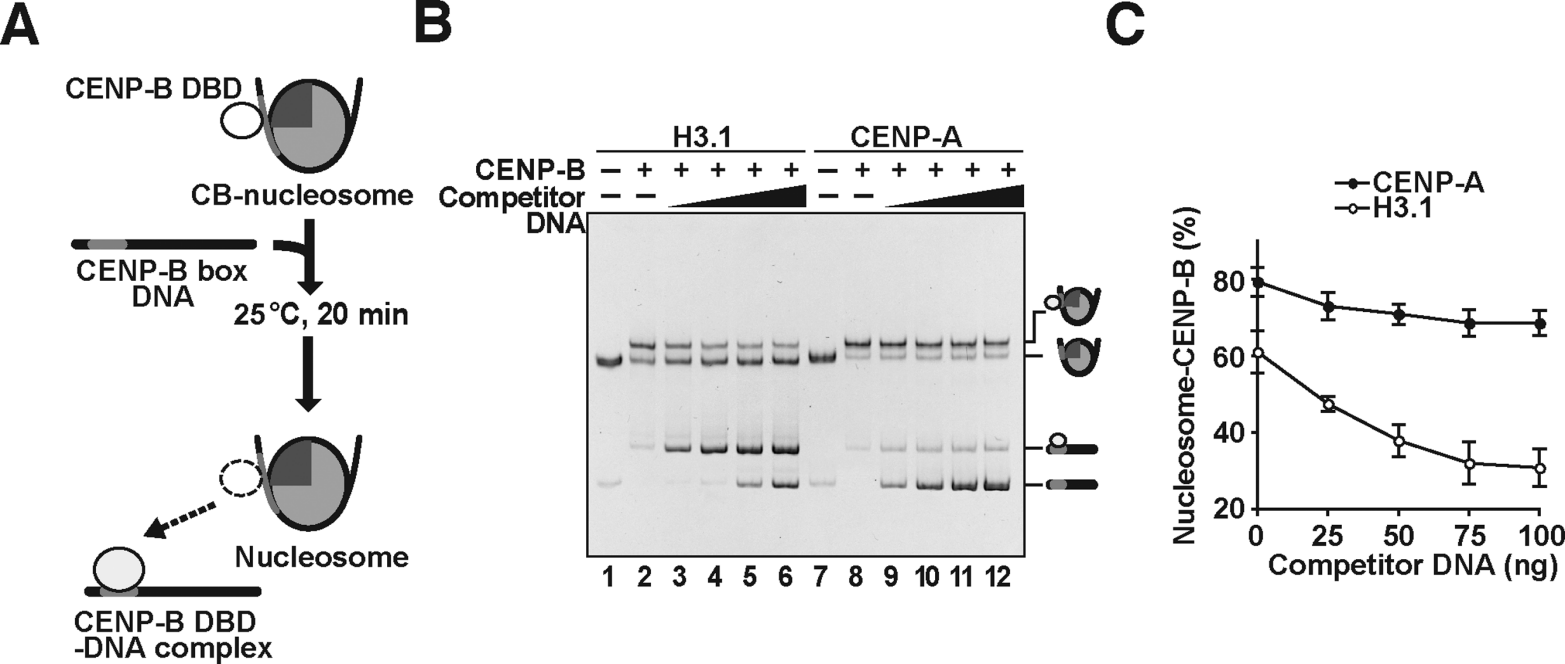
CENP-B binds to the CENP-A nucleosome more stably than the H3.1 nucleosome. (**A**) Schematic representation of the competitive CENP-B retention assay. (**B**) The H3.1 or CENP-A nucleosomes (containing 100 ng DNA) complexed with the CENP-B DBD were incubated in the presence of a naked 166 base-pair DNA containing the CENP-B box sequence. Lanes 1–6 and lanes 7–12 indicate the experiments with the H3.1 and CENP-A nucleosomes, respectively. The amounts of naked 166 base-pair DNA are 0 ng (lanes 2 and 8), 25 ng (lanes 3 and 9), 50 ng (lanes 4 and 10), 75 ng (lanes 5 and 11), and 100 ng (lanes 6 and 12). Lanes 1 and 7 indicate control experiments without the CENP-B DBD and the naked 166 base-pair DNA. (**C**) Graphic representation of the experiments shown in panel (B). The amounts (%) of nucleosomes complexed with the CENP-B DBD were plotted against the amounts of competitor DNA. Averages of four independent experiments are shown with standard deviation values.

We next compared the stabilities of the CA-CB and H3.1-CB nucleosomes. To do so, we titrated the CA-CB and H3.1-CB nucleosomes with increasing amounts of the naked 166 base-pair DNA fragment with the CENP-B box, and evaluated the CENP-B DBD dissociation from the CA-CB and H3.1-CB nucleosomes (Figure 2A–C). Upon DNA titration, the CENP-B DBD dissociated from the H3.1-CB nucleosome, and the amounts of the CENP-B DBD-DNA complex and the H3.1 nucleosome without the CENP-B DBD increased in a DNA concentration-dependent manner (Figure 2B, lanes 1–6, and 2C). In contrast, the CA-CB nucleosome stably existed in the presence of an excess amount of the DNA (Figure 2B, lanes 7–12, and 2C). These results suggested that CENP-B associates more stably with the CENP-A nucleosome than the H3.1 nucleosome.

### CENP-B binds more stably to the proximal entry/exit sites of the CENP-A nucleosome

**Figure 3.**
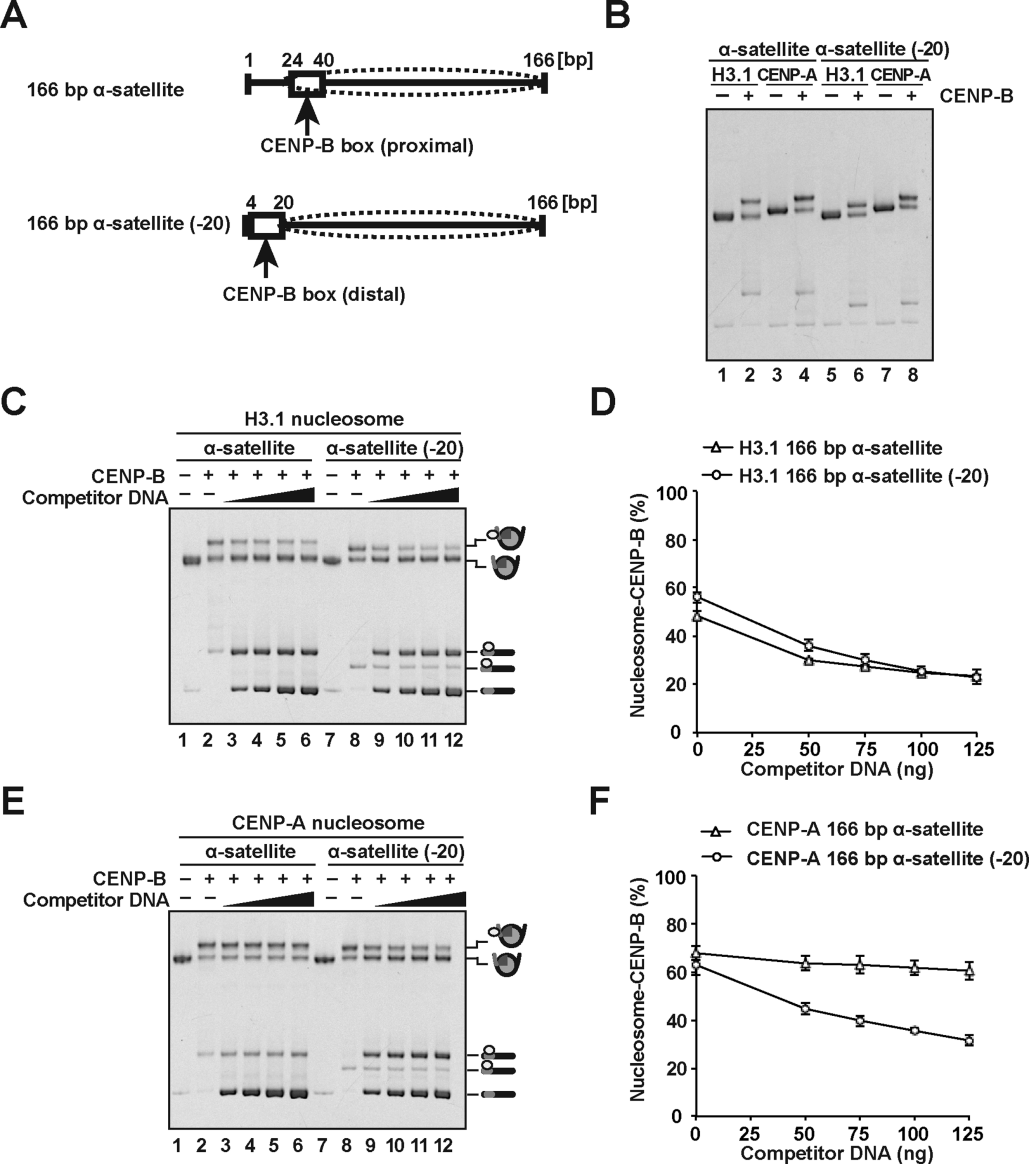
CENP-B binds more stably to the proximal DNA region of the CENP-A nucleosome. (**A**) Schematic representation of the proximal and distal CENP-B box locations, relative to the CENP-A nucleosome (dotted ellipses). The upper and lower panels illustrate the nucleosomes with the 166 base-pair α-satellite DNA (used in Figures 1 and 2) and the 166 base-pair α-satellite (-20) DNA, respectively. (**B**) Electrophoretic mobility shift assay. The H3.1 and CENP-A nucleosomes (lanes 1, 3, 5 and 7) and those complexed with the CENP-B DBD (lanes 2, 4, 6 and 8, respectively) were analyzed by non-denaturing 6% polyacrylamide gel electrophoresis with ethidium bromide staining. Lanes 1, 2, 5 and 6 indicate the H3.1 nucleosomes, and lanes 3, 4, 7 and 8 indicate the CENP-A nucleosomes. Lanes 1–4 and lanes 5–8 are experiments with the 166 base-pair α-satellite DNA and the 166 base-pair α-satellite (-20) DNA, respectively. (**C**) The H3.1 (containing 100 ng DNA) complexed with the CENP-B DBD were incubated in the presence of the naked 166 base-pair α-satellite DNA. The amounts of naked 166 base-pair α-satellite DNA are 0 ng (lanes 2 and 8), 50 ng (lanes 3 and 9), 75 ng (lanes 4 and 10), 100 ng (lanes 5 and 11) and 125 ng (lanes 6 and 12). Lanes 1 and 7 indicate control experiments without the CENP-B DBD and the naked 166 base-pair DNA. (**D**) Graphic representation of the experiments shown in panel (C). The amounts (%) of H3.1 nucleosomes complexed with CENP-B DBD were plotted against the amounts of competitor DNA. Averages of three independent experiments are shown with standard deviation values. (**E**) The CENP-A nucleosomes (containing 100 ng DNA) complexed with the CENP-B DBD were incubated in the presence of the naked 166 base-pair α-satellite DNA. The amounts of naked 166 base-pair α-satellite DNA are 0 ng (lanes 2 and 8), 50 ng (lanes 3 and 9), 75 ng (lanes 4 and 10), 100 ng (lanes 5 and 11) and 125 ng (lanes 6 and 12). Lanes 1 and 7 indicate control experiments without the CENP-B DBD and the naked 166 base-pair DNA. (**F**) Graphic representation of the experiments shown in panel (E). The amounts (%) of CENP-A nucleosomes complexed with CENP-B DBD were plotted against the amounts of competitor DNA. Averages of three independent experiments are shown with standard deviation values.

We next tested the CENP-B binding to the distal linker region of the nucleosomes. To do so, we prepared the CA-CB (-20) and H3.1-CB (-20) nucleosomes (Figure 3A and B). In these nucleosome samples, the CENP-B DBD bound to the CENP-B box sequence located at the distal linker DNA region, which is not directly wrapped around the histone core (Figure 3A). Interestingly, in the H3.1 nucleosome, the CENP-B retention ability was unaffected, when the CENP-B box was moved to the distal linker region, as in the H3.1-CB (-20) nucleosome (Figure 3C and D). However, surprisingly, the CA-CB (-20) nucleosome substantially reduced the CENP-B retention ability, as compared to that of the CA-CB nucleosome (Figure 3E and F). These results strongly suggest that CENP-B binds more tightly to the proximal region than the distal linker region in the CENP-A nucleosome, but not in the H3.1 nucleosome. Therefore, the specific interaction between CENP-B and the CENP-A nucleosome may enhance the stability of CA-CB nucleosome formation.

### The CENP-B DBD specifically interacts with the CENP-A-H4 complex

**Figure 4.**
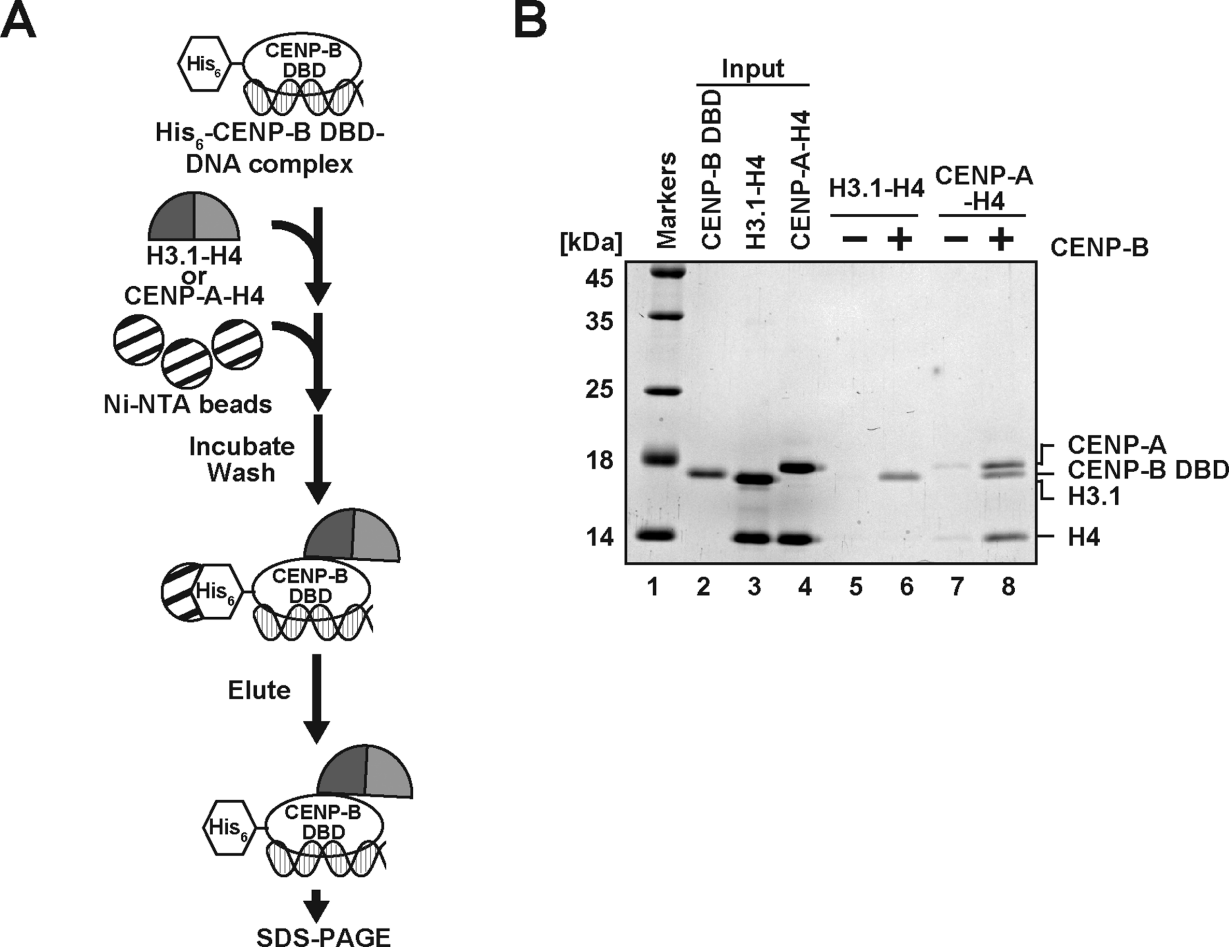
The CENP-B DBD specifically binds to the CENP-A-H4 complex. (**A**) Schematic representation of the pull-down assay with the His_6_-tagged CENP-B DBD and the CENP-A-H4 or H3.1-H4 complex. (**B**) The His_6_-tagged CENP-B DBD (50 nM) complexed with a 21 base-pair DNA was incubated with the CENP-A-H4 or H3.1-H4 complex (50 nM). The proteins pulled down with the Ni-NTA agarose beads were analyzed by 16% SDS-PAGE with Coomassie Brilliant Blue staining. Lane 1 indicates the molecular mass markers. Lane 2: His_6_-tagged CENP-B DBD (50% of input). Lane 3: the H3.1-H4 complex (20% of input). Lane 4: the CENP-A-H4 complex (20% of input). Lanes 5 and 6 represent the pull-down experiments with the H3.1-H4 complex, in the absence and presence of the His_6_-tagged CENP-B DBD, respectively. Lanes 7 and 8 represent the pull-down experiments with the CENP-A-H4 complex, in the absence and presence of the His_6_-tagged CENP-B DBD, respectively.

We then tested whether the CENP-B DBD directly interacts with CENP-A. To do so, we prepared the CENP-A-H4 and H3.1-H4 complexes, and performed pull-down assays with the His_6_-tagged CENP-B DBD (Figure 4A). Since CENP-B DBD alone does not properly fold without its target DNA (41,51), we used the His_6_-tagged CENP-B DBD complexed with a 21 base-pair DNA for the pull-down assay. As shown in Figure 4B (lanes 6 and 8), the His_6_-tagged CENP-B DBD-DNA complex was pulled down with the Ni-NTA agarose beads. We found that CENP-A and H4 were substantially detected in the Ni-NTA agarose-bound fraction with the His_6_-tagged CENP-B DBD-DNA complex (Figure 4B, lane 8). However, CENP-A and H4 were not detected without the His_6_-tagged CENP-B DBD-DNA complex (Figure 4B, lane 7). Since the bands corresponding to the His_6_-tagged CENP-B DBD and H3.1 were overlapped, the CENP-B DBD-DNA complex binding to the H3.1-H4 complex was monitored with the H4 band. We thus found that the CENP-B DBD-DNA complex bound weakly to the H3.1-H4 complex (Figure 4B, lanes 5 and 6). The CENP-A-H4 complex binding may not be mediated by the 21 base-pair DNA, because hardly any H3.1-H4 complex binding to the CENP-B DBD-DNA complex was detected (Figure 4B, lanes 5 and 6). Therefore, these results indicate that the CENP-B DBD specifically binds to the CENP-A-H4 complex, but not the H3.1-H4 complex.

### The CENP-A targeting domain (CATD) of CENP-A is not involved in CENP-B binding to the CENP-A nucleosome

**Figure 5.**
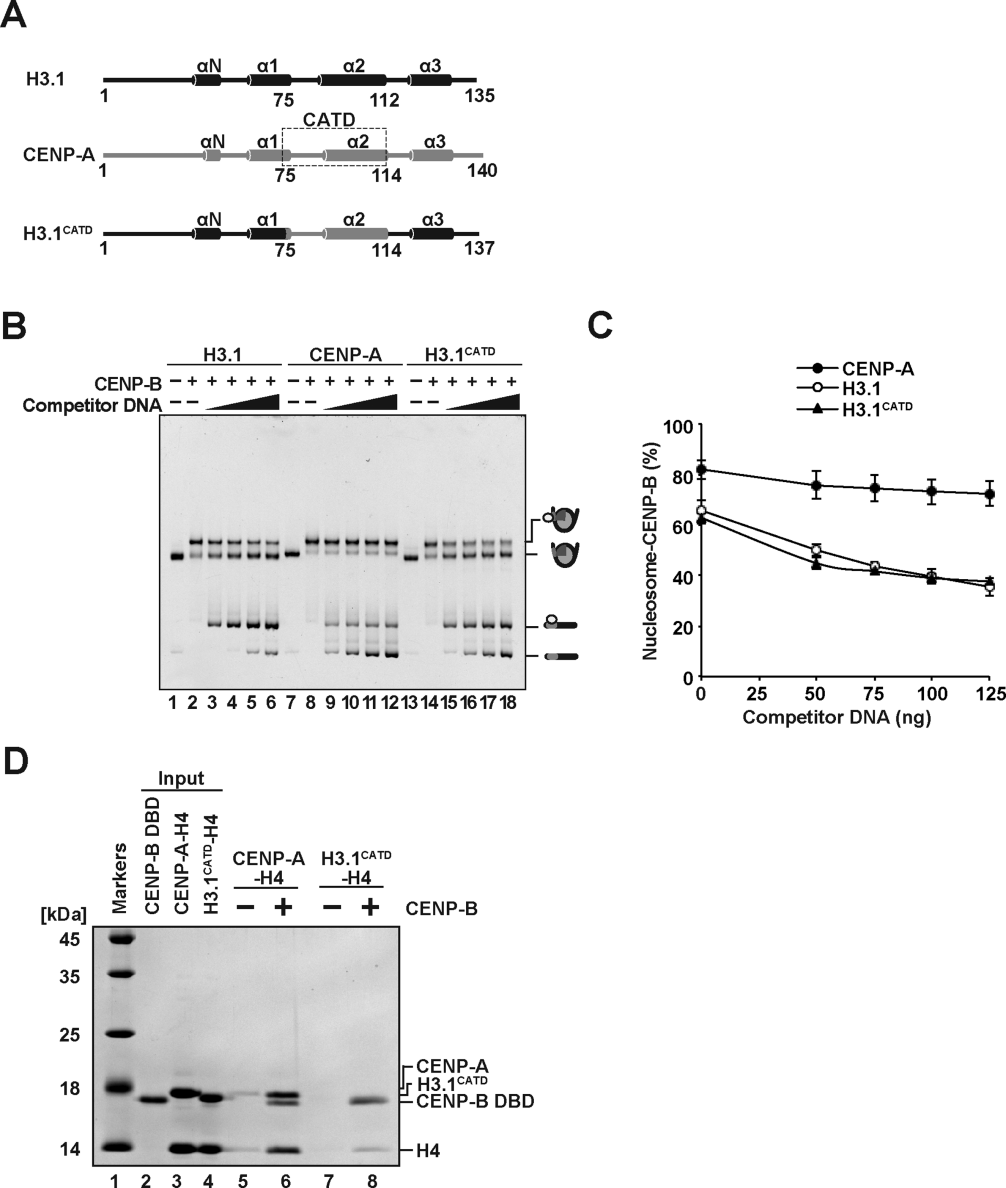
The CENP-B DBD does not stably bind to the H3.1^CATD^ nucleosome. (**A**) Schematic representation of H3.1, CENP-A and H3.1^CATD^. Cylinders indicate the regions that form an α-helix structure in nucleosomes. (**B**) The H3.1, CENP-A, or H3.1^CATD^ nucleosomes (containing 100 ng DNA) complexed with the CENP-B DBD were incubated in the presence of a naked 166 base-pair α-satellite DNA containing the proximal CENP-B box sequence. Lanes 1–6, 7–12 and 13–18 indicate the experiments with the H3.1, CENP-A and H3.1^CATD^ nucleosomes, respectively. The amounts of naked 166 base-pair α-satellite DNA concentrations are 0 ng (lanes 2, 8 and 14), 50 ng (lanes 3, 9 and 15), 75 ng (lanes 4, 10 and 16), 100 ng (lanes 5, 11 and 17) and 125 ng (lanes 6, 12 and 18). Lanes 1, 7 and 13 indicate control experiments without the CENP-B DBD and the naked 166 base-pair DNA. (**C**) Graphic representation of the experiments shown in panel (B). Averages of four independent experiments are shown with standard deviation values. (**D**) The pull-down assay with the H3.1^CATD^-H4 complex. The experiments were performed as in Figure 4B. Lane 1 indicates the molecular mass markers. Lane 2: His_6_-tagged CENP-B DBD (50% of input). Lane 3: the CENP-A-H4 complex (20% of input). Lane 4: the H3.1^CATD^-H4 complex (20% of input). Lanes 5 and 6 represent the pull-down experiments with the CENP-A-H4 complex, in the absence and presence of the His_6_-tagged CENP-B DBD, respectively. Lanes 7 and 8 represent the pull-down experiments with the H3.1^CATD^-H4 complex, in the absence and presence of the His_6_-tagged CENP-B DBD, respectively.

Fachinetti *et al*. reported that the CENP-A N-terminal tail may function in the CENP-B loading at centromeres (45). To test whether the CENP-B DBD actually interacts with the CENP-A N-terminal region, we prepared nucleosomes containing the H3.1^CATD^ mutant (52), which replaces the CENP-A N-terminal tail and the αN helix regions with the corresponding H3.1 regions, but contains the L1 loop and the α2 helix of CENP-A (Figure 5A). The H3.1^CATD^ mutant possesses the centromere-targeting activity in human cells (53).

We then prepared the H3.1^CATD^ nucleosome complexed with the CENP-B DBD (H3.1^CATD^-CB nucleosome) (Figure 1B, lane 6, and 1C, lane 7). The DNA titration experiment revealed that the CENP-B DBD dissociated from the H3.1^CATD^-CB nucleosome (Figure 5B, lanes 13–18 and 5C), as well as the H3.1-CB nucleosome (Figure 5B, lanes 1–6, and 5C). Reproducibly, the CENP-B DBD stably associated with the CENP-A nucleosome in the presence of excess naked DNA (Figure 5B, lanes 7–12, and 5C). These results suggested that the CENP-A N-terminal region is responsible for the stable accommodation of the CENP-B DBD within the nucleosome. Consistent with this idea, we found that the H3^CATD^-H4 complex exhibited significantly weaker binding affinity to the CENP-B DBD, as compared to the CENP-A-H4 complex (Figure 5D). In the pull-down experiment with the H3^CATD^-H4 complex, about 4% of H4 was detected in the CENP-B-bound fraction, as compared to the amount of H4 bound to the CENP-A-H4 complex (Figure 5D).

### CENP-A nucleosome preassembly on both transfected wild type and mutant CENP-B box alphoid DNAs in cells

**Figure 6.**
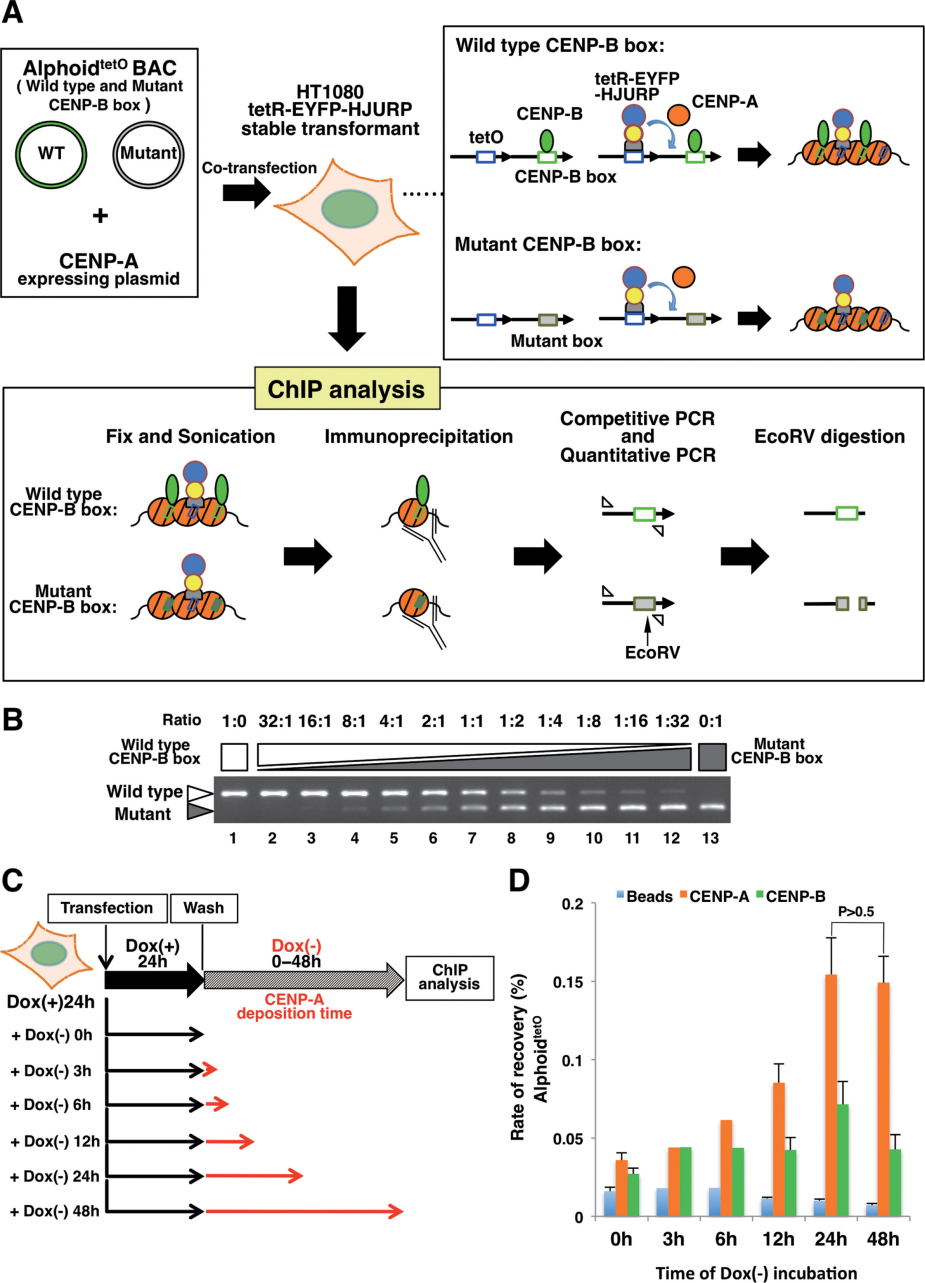
The CENP-A nucleosome preassembly level reaches a maximum after 24h under HJURP tethering conditions. (**A**) Schematic diagram of the CENP-A nucleosome preassembly and the ChIP real-time PCR/competitive PCR analysis. HT1080 cells stably expressing tetR-EYFP-HJURP were transfected with 50 kb alphoid^tetO^ BAC DNAs and Halo-CENP-A expressing plasmid DNA. To generate CENP-A nucleosome preassembly on the transfected alphoid^tetO^ DNAs by the tethering of tetR-EYFP-HJURP, the cells were cultured in doxycycline-free medium. The CENP-A nucleosome assembly levels were analyzed by a ChIP assay. The DNA samples recovered by ChIP were quantitated by real-time PCR. Then, the PCR products (competitively amplified wild type and mutant CENP-B box alphoid^tetO^ DNAs) were digested with EcoRV and analyzed by agarose gel electrophoresis (43,67). (**B**) Example of a competitive PCR control. The wild type and mutant CENP-B box alphoid^tetO^ BAC DNAs were mixed at different ratios, and amplified competitively with the same primer set by PCR. The white arrowhead indicates the PCR fragment from the wild type CENP-B box alphoid^tetO^ DNA. The gray arrowhead indicates the PCR fragment from the mutant CENP-B box alphoid^tetO^ DNA. (**C**) Schematic diagram of the CENP-A deposition and the transient ChIP assay. The cells transfected with the alphoid^tetO^ BAC DNA mixture were cultured with medium containing doxycycline for 24 hr. To analyze CENP-A nucleosome deposition at tetO sites on the transfected alphoid^tetO^ DNAs, the cells were cultured with doxycycline-free medium for 0–48 hr. The ChIP assay was then performed with anti-CENP-A and anti-CENP-B antibodies. The relative copy number of the total alphoid^tetO^ array was quantitated by real-time PCR. The black line indicates the culture with medium containing doxycycline. The red line indicates the culture with doxycycline-free medium. (**D**) CENP-A preassembly levels on the total alphoid^tetO^ DNAs, determined by the ChIP analysis. The relative copy numbers of the total alphoid^tetO^ array recovered by the beads with the anti-CENP-A antibody or anti-CENP-B antibody, or without antibody, were quantitated by real-time PCR, and are displayed as graphs. Error bars represent the SEM (n = 3). *P*-values obtained with the *t*-test are indicated.

Our previous analyses indicated that CENP-B binding to the CENP-B box is required for *de novo* CENP-A nucleosome assembly and functional centromere formation, as a stable human artificial chromosome (HAC) on the input alphoid DNA (43,67,68). However, the CENP-B-dependent *de novo* CENP-A assembly was only observed after several days of time lag after the alphoid DNA transfection. In the present study, we next compared the CENP-A nucleosome stability in the presence or absence of CENP-B binding to the CENP-B box on alphoid DNA in cells. For this purpose, we developed a protocol applying chromatin immunoprecipitation (ChIP) and quantitative/competitive PCR to evaluate the CENP-A nucleosome stability in cells, in combination with an HJURP tethering system (Figure 6A).

**Figure 7.**
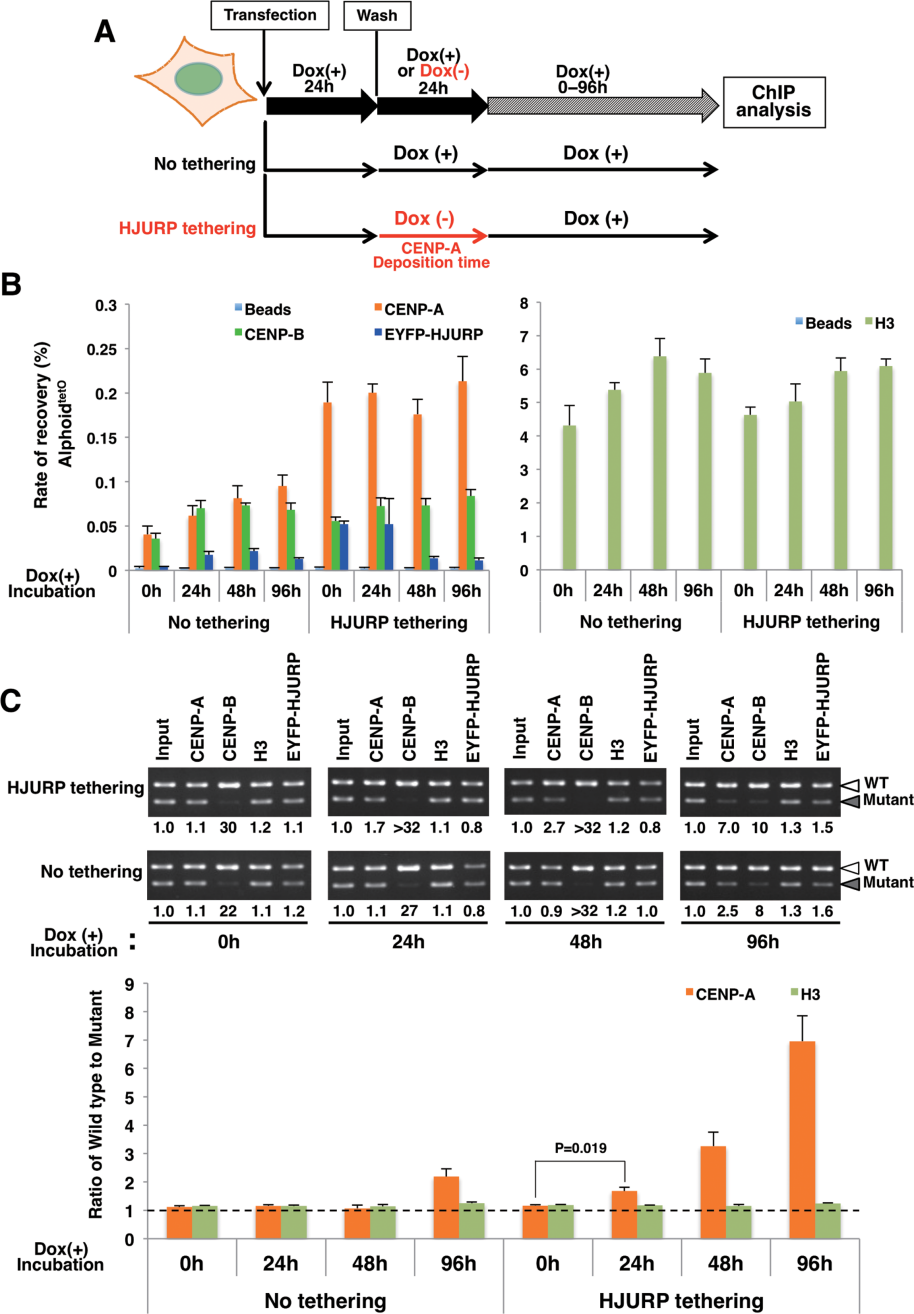
CENP-B binding to alphoid DNA enhances CENP-A retention on nucleosomes. (**A**) Schematic diagram of the CENP-A preassembly followed by the ChIP assay. After co-transfection with the wild type and mutant CENP-B box alphoid^tetO^ DNAs, and a Halo-CENP-A expressing plasmid, the cells expressing tetR-EYFP-HJURP were cultured in medium containing doxycycline for 24 hr. To deposit the maximum level of CENP-A nucleosomes by the tethering of tetR-EYFP-HJURP at tetO sites on the transfected alphoid^tetO^ DNAs, the cells were cultured with doxycycline-free medium for 24 hr. Then, doxycycline was added to the medium to quench the CENP-A deposition. The cells were harvested after 0, 24, 48 and 96 hr of culture in medium containing doxycycline. The ChIP assay and the quantitative PCR/competitive PCR were then performed. The black line indicates the culture with medium containing doxycycline. The red line indicates the culture with doxycycline-free medium, to deposit CENP-A. (**B**) The relative copy number of the total alphoid^tetO^ array, quantitated by real-time PCR. The ChIP assay was performed with anti-CENP-A, anti-CENP-B, anti-histone H3, and anti-GFP (also recognizing EYFP) antibodies. The bars show the relative rates of recovery of the total alphoid^tetO^ DNA against the input DNA. Error bars represent the SEM (n = 3). (**C**) DNA samples recovered after the ChIP assay were analyzed by competitive PCR. The relative enrichment of the wild type CENP-B box alphoid^tetO^ DNA versus the mutant CENP-B box alphoid^tetO^ DNA is shown below the gel images. White arrowheads indicate the PCR fragments from the wild type CENP-B box alphoid^tetO^ DNA. Gray arrowheads indicate the PCR fragments from the mutant CENP-B box alphoid^tetO^ DNA. The values of CENP-A and H3 are normalized by the input DNA. Error bars represent the SEM (n = 3). P-values obtained with the t-test are indicated.

We generated preassembled CENP-A nucleosomes on transfected wild type and mutant CENP-B box alphoid DNAs equally in HT1080 cells. tetR-EYFP-HJURP was tethered on the co-transfected alphoid^tetO^ bacterial artificial chromosome (BAC) DNA mixture containing equal amounts of wild type and mutant CENP-B boxes (1:1). HJURP is known as a prominent CENP-A chaperone, and it robustly promotes CENP-A assembly in chromosomes (54,55). Exogenous CENP-A expression from the co-transfected Halo-CENP-A expression plasmid enhances the CENP-A deposition by tetR-EYFP-HJURP tethering within a shorter period. These alphoid^tetO^ DNAs are based on the synthetic alphoid dimer unit, in which one monomer contains a CENP-B box or mutant CENP-B box, and the adjacent monomer contains a tetracycline operator sequence, instead of the counter position for the CENP-B box (Figure 6A)(49). Two point mutations within the canonical CENP-B box sequence in the alphoid DNA abolish the specific CENP-B binding. The total amounts of wild type and mutant CENP-B box alphoid DNAs were quantitated by ChIP and real-time PCR analyses, and the ratios of the wild type and mutant CENP-B box alphoid DNAs were quantitated by a competitive PCR method (Figure 6A and B). Under the experimental conditions used in this assay, the CENP-A deposition level on the alphoid^tetO^ DNA, detected by ChIP and quantitative PCR analyses, reached almost a maximum level within 24 hr after the initiation of the tetR-HJURP tethering (Figure 6C and D). Although CENP-B was specifically enriched on the wild type CENP-B box DNA, no biased enrichment of CENP-A was detected between the wild type and mutant CENP-B box alphoid^tetO^ DNAs after 24 hr of tetR-HJURP tethering (see Figure 7C Dox(+) 0h). Thus, we chose 24 hr for the tetR-HJURP tethering period, to achieve a high level of CENP-A nucleosome preassembly.

### Binding of CENP-B to the CENP-B box enhances the retention of the preassembled CENP-A nucleosome on alphoid DNA *in vivo*

We then examined whether CENP-B binding to the CENP-B boxes stabilizes the CENP-A nucleosomes on input alphoid DNA *in vivo*, in the actual ChIP experiments. After a 24 hr CENP-A preassembly period, doxycycline was added to the medium to prevent the continuous deposition of CENP-A on the alphoid^tetO^ DNAs (Figure 7A). After 0 hr, 24 hr, 48 hr and 96 hr incubation times, we analyzed the preassembled CENP-A levels in the presence or absence of CENP-B binding, by ChIP and quantitative/competitive PCR assays (Figure 7B and C). The recovery rate of the total alphoid^tetO^ DNAs with the anti-CENP-A antibody revealed that similar high levels of CENP-A assembly continued during 0 ∼ 96 hr of culture with doxycycline, as determined by the quantitative PCR assay (Figure 7B). However, the competitive PCR experiments clearly showed that, during these cultures, the CENP-A nucleosome gradually became preferentially enriched on the wild type CENP-B box DNA, as compared to the mutant CENP-B box DNA (Figure 7C, 1.1-, 1.7-, 2.7- and 7.0-fold for 0 hr, 24 hr, 48 hr and 96 hr, respectively). These results strongly suggested that the CENP-A nucleosome is more stably retained on the wild type CENP-B box DNA associated with CENP-B, than on the mutant CENP-B box DNA. The recovery rate of the total alphoid^tetO^ DNAs is lower with anti-CENP-B than that with anti-CENP-A, especially under the tetR-HJURP tethering conditions (Figure 7B). This result is consistent with the ability of CENP-B to bind only to the wild type CENP-B box alphoid^tetO^ DNA.

Without the CENP-A preassembly conditions (Figure 7B and C, no HJURP tethering), the recovery rate of the total alphoid^tetO^ DNAs with the anti-CENP-A antibody was low. Thus, non-biased assembly between the wild type and mutant CENP-B box DNAs occurred in the culture until 48 hr, and the initial biased assembly of CENP-A was detected at 96 hr on the wild type CENP-B box DNA (2.5-fold). This result is consistent with our previous analysis showing that the CENP-B-dependent *de novo* CENP-A assembly in HT1080 cells was detectable on the third or fourth day after DNA transfection (43). During these cultures, the H3 nucleosome assembled without bias on the wild type and mutant CENP-B box DNAs (Figure 7B and C). In addition, at least during the several initial days after DNA transfection, the majority of the naked DNAs introduced into the HT1080 cells cannot replicate efficiently (data not shown). This is consistent with the present observation that no major replicative dilution of the preassembled CENP-A nucleosomes was observed, when the binding of the tetR-HJURP was prevented by adding doxycycline (at 24–48 hr in Figure 7B, before the CENP-B-dependent *de novo* CENP-A assembly started at 96 hr in Figure 7B). *De novo* artificial chromosome formation accompanying self-propagation activity may also be established after (or be associated with) the CENP-B-dependent *de novo* CENP-A assembly (43).

Thus, all of these experiments clearly demonstrated that, once assembled, the CENP-A nucleosomes were stabilized on the alphoid DNA along with CENP-B binding to the CENP-B box in HT1080 cells.

## DISCUSSION

The CENP-B box sequence is preferentially positioned near the entry/exit DNA sites of the CENP-A nucleosome *in vitro* and *in vivo* (15,23,25,42). This fact prompted us to test whether CENP-B preferentially binds to the CENP-A nucleosome, as compared to the canonical H3 nucleosome. However, no obvious difference in the CENP-B binding was detected between the CENP-A and H3.1 nucleosomes (Figure 1B)(46). This suggested that the CENP-B accessibility to the CENP-B box sequence located near the entry/exit sites may not be substantially different between the CENP-A and H3.1 nucleosomes.

To address the mechanism by which CENP-B binds to the CENP-A nucleosome, in the present study, we prepared the CENP-A and H3.1 nucleosomes complexed with the CENP-B DBD, and evaluated the CENP-B retention upon titration with a competitor DNA. We found that the CENP-B DBD was more stably retained in the CENP-A nucleosome than the H3.1 nucleosome (Figure 2B and C). These results support the conclusion that CENP-B is somehow specifically accommodated on the CENP-A nucleosome. Surprisingly, we found that CENP-B was retained more stably at the proximal region of the CENP-A nucleosome than the distal linker region (Figure 3). This suggests the existence of a specific interaction between CENP-B and the CENP-A nucleosome. Consistent with this idea, we eventually detected the specific interaction between the CENP-B DBD and CENP-A (Figure 4). The N-terminal tail region of CENP-A is reportedly required for the proper centromere loading of CENP-B in cells (45). Interestingly, the CENP-B retention was significantly reduced in the H3^CATD^ nucleosome, as compared to the CENP-A nucleosome (Figure 5). H3^CATD^ contains the CENP-A-targeting domain of CENP-A (52,53), but not the CENP-A αN helix and N-terminal tail. Our pull-down assays also revealed that CENP-B binding was substantially lower with the H3^CATD^-H4 complex than the CENP-A-H4 complex (Figure 5). Therefore, the CENP-B DBD may interact with the N-terminal region of CENP-A, and form a stable CA-CB nucleosome.

The CENP-B DBD (amino acid residues 1–129) used in the present *in vitro* study constitutes a basic domain (pI = 10.5), although the entire CENP-B (599 amino acid residues) is an acidic protein (31). We suppose that the specific binding of the CENP-B DBD to the CENP-A nucleosome is functional in cells, because the previous *in vivo* data showed that the CENP-B DBD itself induces *de novo* CENP-A assembly on human alphoid DNA in cells (43). Although the full-length CENP-B has not been tested *in vitro* yet, the *in vivo* experiments shown in Figures 6 and 7 revealed that the full-length CENP-B also forms a stable complex with the CENP-A nucleosome. Therefore, the specific interaction between the CENP-B DBD and CENP-A described here may be important for the formation of a stable CA-CB nucleosome in functional centromeres.

In addition to CENP-B, other centromere proteins, CENP-C, CENP-S, CENP-T, CENP-W and CENP-X, are also DNA-binding proteins that specifically accumulate in the centromeric chromatin (34–40). Unlike CENP-B, these centromeric DNA-binding proteins do not exhibit unequivocal DNA sequence binding specificity. In the crystal structure of the human CENP-A nucleosome, the DNA ends are disordered because of their flexible nature (15). Consistently, small angle X-ray scattering and biochemical analyses revealed that the DNA ends of the nucleosomes containing CENP-A (or its homologs) are also flexible (16–20). These facts suggest that the flexible nature of the DNA in the CENP-A nucleosome is evolutionarily conserved, and may play an important role in the centromeric chromatin architecture. The DNA end flexibility of the CENP-A nucleosome may also function to regulate the DNA binding and/or retention of these centromeric DNA-binding proteins in centromeres. Further studies are awaited.

The CENP-A nucleosome itself may be unstable *in vitro*, probably due to the detached DNA at both ends (15–20,56). This suggests that an additional factor(s) must be required for the stable maintenance of the CENP-A nucleosome *in vivo*. In the present study, the CENP-A nucleosome was stably maintained *in vivo* in the vicinity of the CENP-B binding site (Figure 7). Intriguingly, the CENP-A nucleosome became substantially unstable in the absence of the CENP-B box sequence in human cells (Figure 7). Therefore, CENP-B may indeed function as one of the factors that compensate for the instability of the CENP-A nucleosome in the centromeric chromatin on the alphoid DNA, especially in the initial *de novo* CENP-A assembly stage (before CENP-C assembly).

The CENP-B gene knockout mice are viable (57–59). Thus, CENP-B is either not required for the maintenance of an established centromere or functionally redundant. A recent report indicated that once assembled, the centromere can maintain its function, probably through the CENP-A C-terminal tail to the CENP-C pathway, without CENP-B (45). CENP-C binds the C-terminal peptides of nucleosomal CENP-A (60–62). This can explain the rare phenomenon of the ‘neocentromere’, the formation of a new centromere on a non-centromeric alphoid locus, and the centromere on Y-alphoid DNA, which both lack CENP-B and the CENP-B box. However, significantly lower CENP-A levels were actually observed in human Y-centromeres and neocentromeres (63–65). Moreover, *de novo* centromere and human artificial chromosome (HAC) formation accompanied by *de novo* CENP-A assembly occur only on input alphoid DNA with intact CENP-B boxes (43,66–68). No HAC formation or *de novo* stable CENP-A nucleosome assembly was observed when the CENP-B boxes were mutated or reduced or the CENP-B gene was deleted. Therefore, CENP-B binding to the CENP-B box affects CENP-A nucleosome assembly and/or stability on alphoid DNA, and may function in *de novo* centromere formation.

In previous experiments, CENP-A, -B and -C co-immunoprecipitated with alphoid DNA (69,70), and CENP-B interacted with CENP-C in a yeast two-hybrid analysis (70). The CENP-A N-terminal tail controls kinetochore function through the CENP-B level (45). In the present study, we clearly demonstrated that CENP-B binding to the CENP-B box enhanced the retention of preassembled CENP-A nucleosomes on alphoid DNA. Thus, in normal human and mouse autosomes and the X-chromosome, the kinetochore and centromere functions are strongly linked with the underlying repetitive DNA through the CENP-B box, CENP-B, CENP-A and probably CENP-C. These two possible kinetochore recruiting pathways, from the CENP-A C-terminal tail to CENP-C, and from the CENP-A N-terminal tail to CENP-B, may provide the answer to why the protein required for *de novo* centromere and HAC formation on alphoid DNA is not required for the function and maintenance of the established centromere itself.

The previous findings, together with our present results, strongly suggest that CENP-B binding to the CENP-A nucleosome plays an important role to establish the functional CENP-A nucleosome in mammalian cells. It is also extremely interesting that, in addition to the direct positive effects of CENP-B on CENP-A nucleosome stability, as shown in the present study, CENP-B indirectly destabilizes the CENP-A nucleosome by changing the epigenetic status of the chromatin (by enhancing heterochromatin formation) at the ectopic alphoid integration site (43,49). Further studies are required to clarify the molecular mechanisms controlling the dualistic functions of CENP-B at centromeric and pericentromeric heterochromatin.
